# Nickel phthalocyanine@graphene oxide/TiO_2_ as an efficient degradation catalyst of formic acid toward hydrogen production

**DOI:** 10.1038/s41598-021-95382-z

**Published:** 2021-08-09

**Authors:** Sajjad Keshipour, Shima Mohammad-Alizadeh

**Affiliations:** Department of Nanotechnology, Faculty of Science, Urmia University, Urmia, Iran

**Keywords:** Chemistry, Catalysis, Heterogeneous catalysis

## Abstract

A new photocatalytic system was introduced to degrade formic acid toward hydrogen production using nickel(II) phthalocyanine (NiPc)@graphene oxide (GO)/TiO_2_ as the catalyst. Synthesis of NiPc was performed in the presence of GO leading to a homogeneous distribution of NiPc on GO. While TiO_2_ promoted the reaction using each of NiPc and GO under visible light, the reaction was carried out with superior rate using NiPc@GO/TiO_2_. In this reaction, GO minimized the band gap of TiO_2_ through contributing its Fermi levels and NiPc escalated the photocatalytic reaction rate as a sensitizing agent. The reaction released hydrogen with the rate of 1.38 mmol h^−1^ and TOF = 77 h^−1^.

## Introduction

With increasing concerns about application of fossil fuels, production of hydrogen as a green combustion source has been attracted a great deal of attention^1^. The significance of this subject has led to the introducing various sources such as especial chemicals for the hydrogen production^2^. Although decomposition of most chemicals toward hydrogen production produces toxic wastes, formic acid (FA) degradation is a green approach for this agenda. A great number of catalysts for the FA degradation have been reported in the recent years. Ru as one of the effective catalysts have promoted the reaction in various forms such as [Ru(H_2_O)_6_]^2+^, RuCl_3_^3^, [RuCl_2_(p-cymene)]_2_^4^, and ruthenium carbonyl complexes^5^. Meanwhile, Pt and Ru-loaded CdS/Al-HMS catalyzed FA decomposition under visible light with the reaction rate of 7.63 ml h^−1^^6^. Titanium dioxide modified with various metals and metal oxides has been employed in the FA degradation reaction as photo-oxidation catalysts^7–11^. In a major advance in 2013, N-doped titanium oxide was used as a semiconductor accompany with Au photocatalyst^12^. Palladium nanoparticles immobilized on nitride carbon-coated mesoporous tungsten oxide was also selected to promote this reaction with high conversions in low catalyst loading^13^.

Phthalocyanines (Pc) are considered as one of the most important categories of colorant. Easy production approach and high efficiency have led to the development of new applications in photodynamic therapy, optical data storage, reverse saturable absorbers, and solar screens^14^. In addition, Pcs especially metalo-Pc such as NiPc are known as catalysts for a variety of reactions^15–19^. The structure of NiPc, consisted from a long conjugated Π-system, provides opportunity to trap light in order to promote a photocatalytic reaction. NiPc composites photocatalytically catalyzed degradation reactions of Rhodamine B, and 2,4-dichlorophenol^20, 21^. Rh-complexes/NiPc was employed as efficient water splitting catalyst under visible irradiation^22^. While other composites of Ni like NiO was employed widely as a semiconductor in the H_2_ production, use of NiPc has not been well established in this reaction^23–26^.

**Scheme 1:**
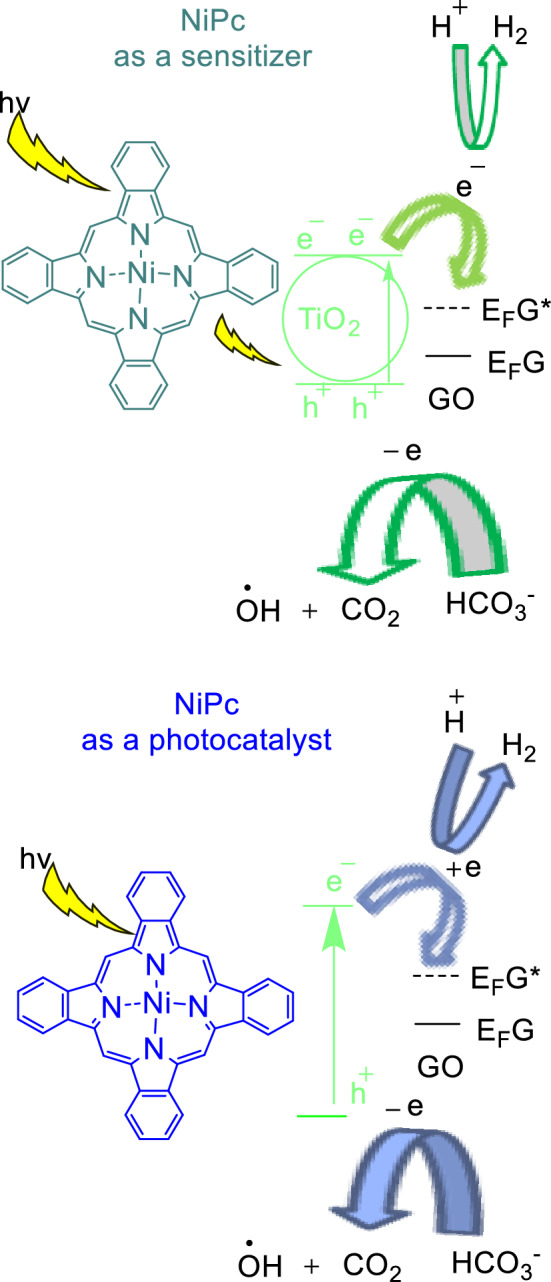
FA degradation by NiPc@GO/TiO_2_.

In continue of our efforts to extend the application of Pcs as a heterogeneous catalyst^27–29^, herein a three-component catalytic system, NiPc@GO/TiO_2_ NPs, efficiently promoted hydrogen production from FA degradation under visible irradiation. In this reaction, GO improves the electron injection efficiency and retards the charge recombination in the semiconductor, TiO_2_^30^. While NiPc as a sensitizer enhances photon interaction with TiO_2_, obtained results showed that it can also act as a semiconductor like TiO_2_. Therefore, two possible pathways are conceivable for degradation of FA in the presence of NiPc@GO/TiO_2_ NPs (Scheme 1).

## Experimental

### Material and methods

All reagents were purchased from Sigma-Aldrich and used without further purification. Anatase form of TiO_2_ with the particle size under 25 nm was purchased from Sigma-Aldrich. FA 85% was provided from Iranico company. Ni determination was carried out on a Flame Atomic Absorption Spectroscopy (FAAS) (Shimadzu 105 model AA-680 atomic absorption spectrometer) with a hollow cathode lamp. Transition electron microscopy (TEM) micrographs were obtained with Philips CM100 BioTWIN transmission electron microscope and TEM Philips EM 208S. Produced gasses were analyzed using an online gas chromatograph (GC-9890A) with thermal conductivity detector (TCD) and flame ionization detector (FID). Gas products (e.g., CO_2_ and H_2_) were detected by TCD equipped TDX-01 column. The powder X-ray diffraction (XRD) pattern was obtained using Bruker, D8 ADVANCE X-ray diffractometer with a Cu-K_α_ radiation source (λ = 1.5406 Å) operating at 40 kV, 40 mA, and a scanning range of 5–80° 2θ, with a 2θ scan step of 0.015° and a step time of 0.2 s. Fourier transform infrared spectroscopy (FTIR) was used to characterize different functional groups of the composite using Jasco 6300 FTIR instrument in the range of 600–4000 cm^−1^. XPS spectra were recorded on a Thermo ESCALAB 250 Xi using monochromatic Al K_α_ radiation (1486.6 eV) with a spot size of 850 µm. The spectra acquisition and processing were carried out using the software Thermo Avantage. The sample was stuck on the sample holder using a double-sided carbon tape and then introduced into the preparation chamber and was degassed until the proper vacuum was achieved. Then it was transferred into the analysis chamber where the vacuum was 9–10 mbar. The analysis was carried out using the following parameters: Pass energy of 20 eV, dwell time 50 ms and the Step size 0.1 eV. UV–Vis spectra were collected by a UV–visible spectrophotometer (Biowave II, Biochrom WPA Ltd., UK), which the solution was prepared by dispersing the photocatalyst in EtOH:H_2_O (70:30) under ultrasonication, and then separation of solids by centrifugation. Turnover Frequency (TOF) was calculated by the following equation:

$$TOF = \frac{{{\text{mmol}}\;of\;produced\;Hydrogen}}{{{\text{mmol}}\;of\;used\;NiPc \times Time \left( {\text{h}} \right)}}.$$

### Preparation of NiPc@GO

Phthalic anhydride (8 mmol), urea (40 mmol), ammonium molybdate (0.005 g), NiCl_2_ (2.1 mmol), and GO (1.0 g) were mixed in a round-bottom flask and then placed in a microwave oven. The reaction vessel was irritated by 900 W instrument for 3 min. After that, the vessel was cooled to room temperature and 50 ml H_2_O was added to it. The mixture was sonicated for 15 min at room temperature, filtered off as purple solid, washed with water (5 × 20 ml), and dried in oven at 80 °C to obtain NiPc@GO (21 w%/79 w%) as a purple solid.

### Typical procedure for the degradation of FA by NiPc@GO/TiO_2_

A 300 W xenon lamp with a glass filter (420 nm) was used as a light source in the photocatalytic degradation of FA. Typically, 0.05 g (18 µmol) of NiPc@GO and 0.04 g TiO_2_ were added into 30 mL of FA. Before irradiation, the mixture was stirred in a dark box for 30 min to disperse the catalyst in the mixture. Next, the mixture was irradiated in a photochemical reaction chamber equipped to a gas collector under continuous stirring condition.

## Results and discussion

**Figure 1:**
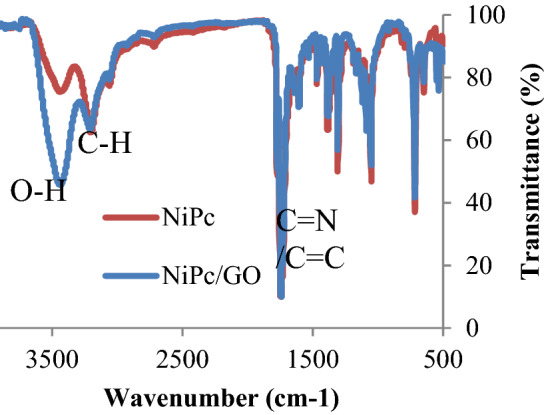
FT-IR spectra of NiPc and NiPc@GO.

FT-IR spectra were prepared for NiPc and NiPc@GO to characterize and compare them (Fig. 1). NiPc’s FT-IR spectrum indicated some absorption bands at 1641, 3103, and 3409 cm^−1^ related to stretching vibrations attributed to C=N/C=C, aromatic C–H, and O–H, respectively. In the meantime, the spectrum of NiPc@GO also showed those peaks just with a little difference for the peak at 3400 cm^−1^ which was appeared in a high intensity due to overlapping of NiPc’s OH peak with that of GO.

**Figure 2:**
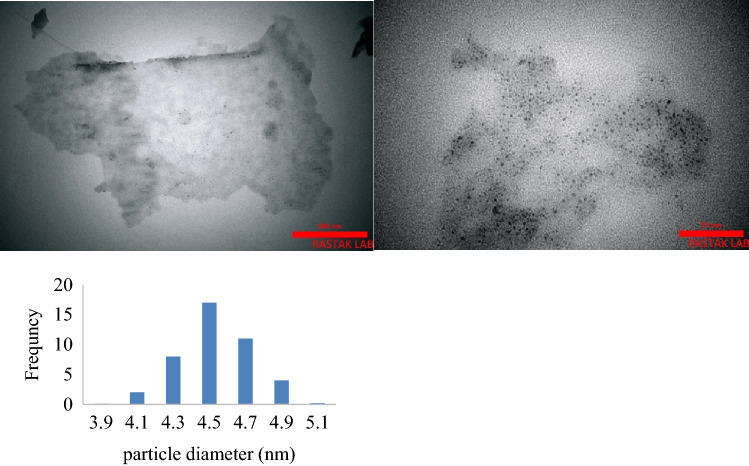
TEM images of NiPc@GO and particle size distribution histogram.

One of the valuable methods to characterize GO composites is TEM which can obviously shows its structure. So, TEM micrographs were prepared for NiPc@GO expecting to see GO layers containing nanoparticles of NiPc (Fig. 2). The micrographs showed wide sheets of GO with homogeneous distribution of NiPc on the GO with the average particle size between 4.1 and 4.9 nm. Since the molecular sizes of MPcs are about 1.5 nm, each of the particles was constructed from aggregation of about 3 molecules. This is very ideal system to obtain very fine particles of a MPc in a heterogeneous system, which finally can run the reaction more effectively.

**Figure 3:**
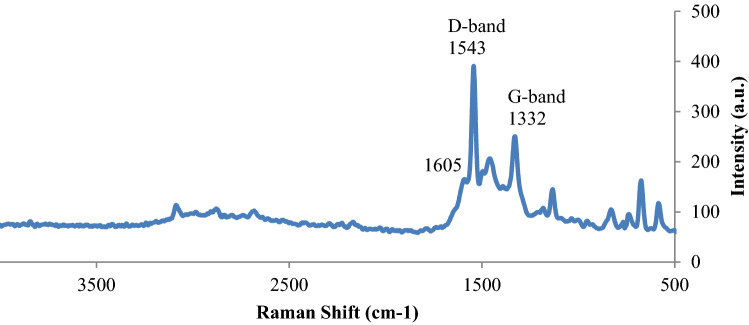
Raman spectrum of NiPc@GO.

The Raman spectroscopy of NiPc@GO was considered for more confirming the structure since GO’s D-band and G-band absorption peaks can be observed there. As can be seen from Fig. 3, the Raman spectrum of NiPc@GO showed D- and G-bands of GO at 1543 and 1332 cm^−1^, respectively. It is crucial to consider that peaks attributed to NiPc appeared in the spectrum such as the peak at 1605 cm^−1^ for C=N band^31^.

**Figure 4:**
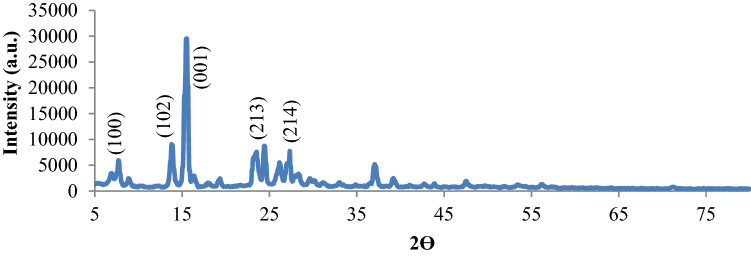
XRD pattern of NiPc@GO.

XRD pattern of NiPc@GO was prepared to determine diffraction peaks related to GO and NiPc. The (001) peak for GO^32^ and (100), (102), (213), and (214) peaks of NiPc^19^ obviously indicated the synthesized nanocomposite structure (Fig. 4). NiPc content of NiPc@GO was determined to be 0.37 mmol per 1 g of the catalyst by FAAS.

**Figure 5:**
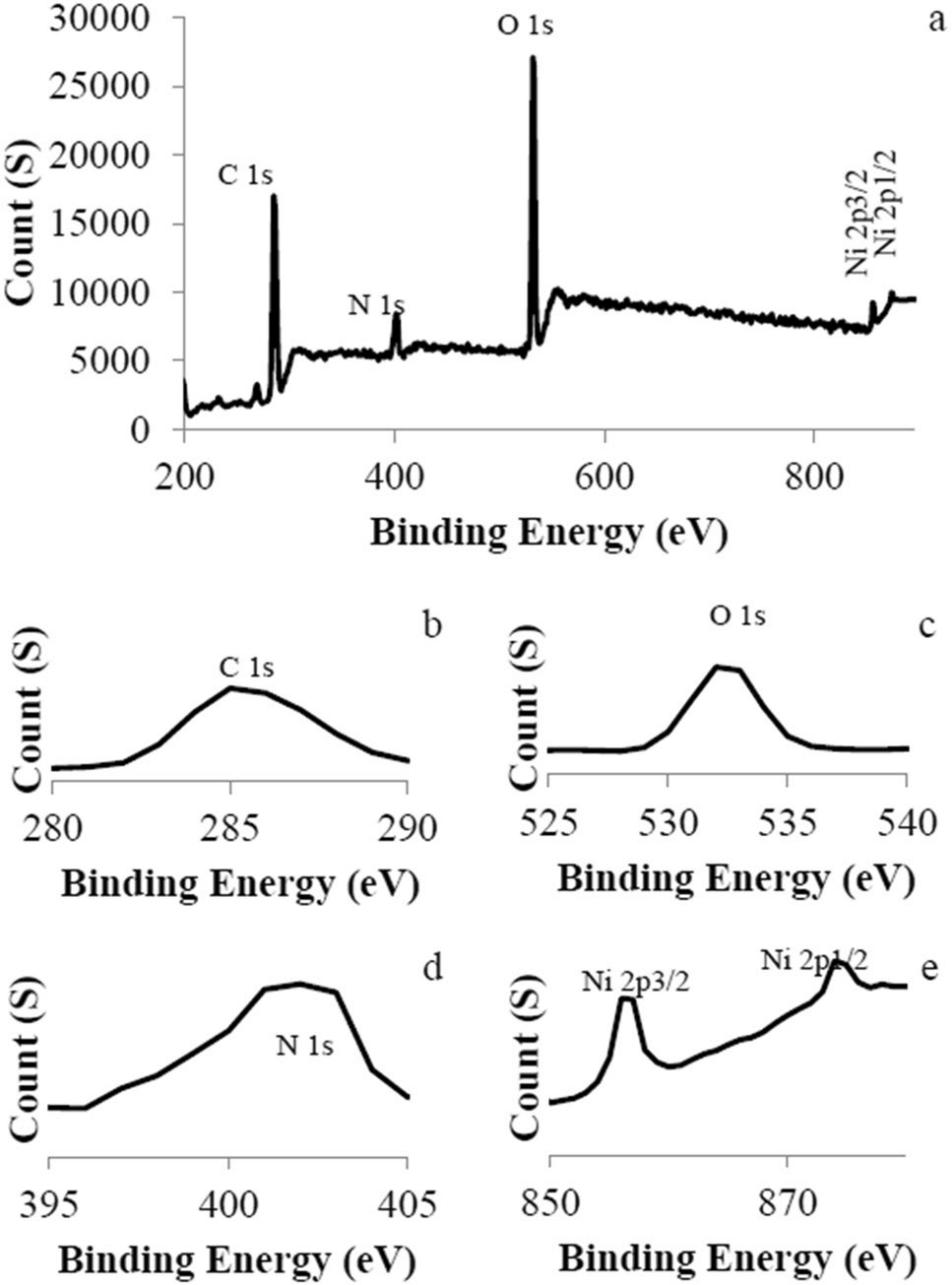
XPS analysis of NiPc@GO.

XPS analysis was performed on NiPc@GO to determine various elements on the nanocomposite (Fig. 5). The analysis showed peaks related to C 1s, N 1s, O 1s, Ni 2p^3/2^, and 2p^1/2^ at 284.9, 402.3, 533.0, 856.1, and 874.3 eV, respectively. The carbon peak at 285.1 eV is the result of overlapping of peaks at 284.4 eV for C=C, 285.7 eV for C–N, and 286.3 eV for C–O bands, which approves presence of two different kinds of structures including Pc and GO. The peaks related to N, and Ni also obviously approve the presence of NiPc in the prepared composite.

**Figure 6:**
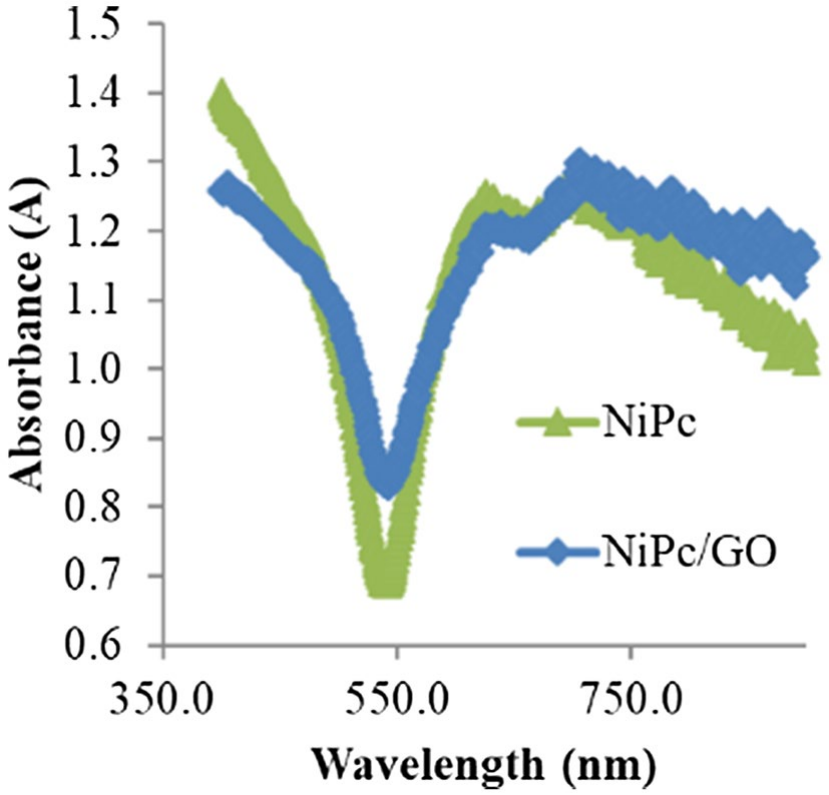
UV–Vis spectra of NiPc and NiPc@GO.

Before starting the photocatalytic reaction using various composites based on NiPc, UV–Vis spectra were prepared for NiPc and NiPc@GO to achieve knowledge about their desired operation wavelength (Fig. 6). Both NiPc and NiPc@GO indicated absorptions under 440 nm and above 460 nm with higher absorption intensity for NiPc@GO. As a result, both NiPc and NiPc@GO should induce high efficiency under visible irradiation for TiO_2_ which is employed as an eminent photocatalyst in the UV region.

The H_2_ production reaction of a dye-sensitized semiconductor composite is usually affected by a series of circumstances such as electron donating system, irradiation wavelength, sensitizing capability and so on^33^. NiPc@GO/TiO_2_ was considered for the H_2_ production since the composite would afford most of the desired conditions for the photocatalytic reaction. Pcs as the potent sensitizers have a conjugated Π system with absorption areas at 620–700 nm known as Q band and about 350 nm known as Soret B band^34^. These absorptions permit to Pcs to have a great ability as a sensitizer in a wide range of wavelengths leading to use of NiPc as a sensitizer in semiconductors^35^. In addition, GO by contribution of its Fermi levels facilitates the electron transportation on TiO_2_ levels as the semiconductor^36^. In this work, the photocatalytic activity of NiPc@GO/TiO_2_ was evaluated in the H_2_ production from FA under Vis irradiation. In the meantime, the effects of various ingredients of the catalyst were investigated on the reaction yield (Table 1).

**Table 1:** H_2_ production by various catalysts from FA degradation reaction.

Entry	Catalyst	H_2_ produced (mmol)		TOF under Vis
		Under Vis	Dark room	
1	TiO_2_	–	–	–
2	GO	–	–	–
3	NiPc	0.61	0.067	34
4	TiO_2_/NiPc	0.63	0.067	35
5	TiO_2_/GO	0.49	–	–
6	NiPc@GO	0.92	0.045	51
7	TiO_2_ (0.03 g)/NiPc (18 µmol)@GO	1.34	0.054	74
8	TiO_2_ (0.04 g)/NiPc (18 µmol)@GO	1.38	0.054	77
9	TiO_2_ (0.05 g)/NiPc (18 µmol)@GO	1.38	0.054	77
10	TiO_2_ (0.04 g)/NiPc (12 µmol)@GO	0.98	0.052	81
11	TiO_2_ (0.04 g)/NiPc (24 µmol)@GO	1.59	0.057	66
12	TiO_2_ (0.04 g)/NiPc (30 µmol)@GO	1.68	0.058	56

Reaction conditions: FA (30 ml), catalyst, r.t., 1 h.

The catalytic performance of NiPc@GO was investigated in the degradation of FA in the presence and absence of TiO_2_ under ambient conditions (Table 1). The results exhibited a high activity for NiPc@GO/TiO_2_ in the degradation reaction toward H_2_ generation under visible light providing 1.38 mmol h^−1^ H_2_ using 18 µmol of the catalyst. H_2_ production was decreased to 0.63 mmol h^−1^ in the presence of NiPc/TiO_2_ under visible irradiation, which approves contribution of GO in the reaction progress. The phenomenon is intelligible considering that GO’s Fermi levels are inserted in TiO_2_ band gaps. This can also be observed from the result of degradation reaction by GO/TiO_2_ with 0.49 mmol h^−1^ H_2_ production rate, while as mentioned the reaction did not proceed using bare TiO_2_. In the meantime, the reaction was examined employing NiPc@GO as the catalyst with 0.92 mmol h^−1^ hydrogen production rate. In spite of the fact that lack of TiO_2_ as a semiconductor should significantly decreases the yield; this negligible decline is not strange when we find out that Pcs are also semiconductor^37^. The lowest amount of TiO_2_ required for obtaining high yield was achieved 0.04 g. The reaction yield was reduced dramatically in a dark room revealing the photo-induced pathway for the reaction. Turnover frequency (TOF) was calculated for the reaction by NiPc@GO/TiO_2_ about 77 h^−1^ which is a high number regarding mild conditions of the reaction. Furthermore, impression of NiPc loading on GO was evaluated on the H_2_ generation rate as well as TOF. 18 µmol of NiPc on GO was recognized as the best catalyst amount regarding both values of the H_2_ production rate and TOF. In all tests, only signals assigned to H_2_ and CO_2_ were detected by gas chromatography without any CO signal.

**Figure 7:**
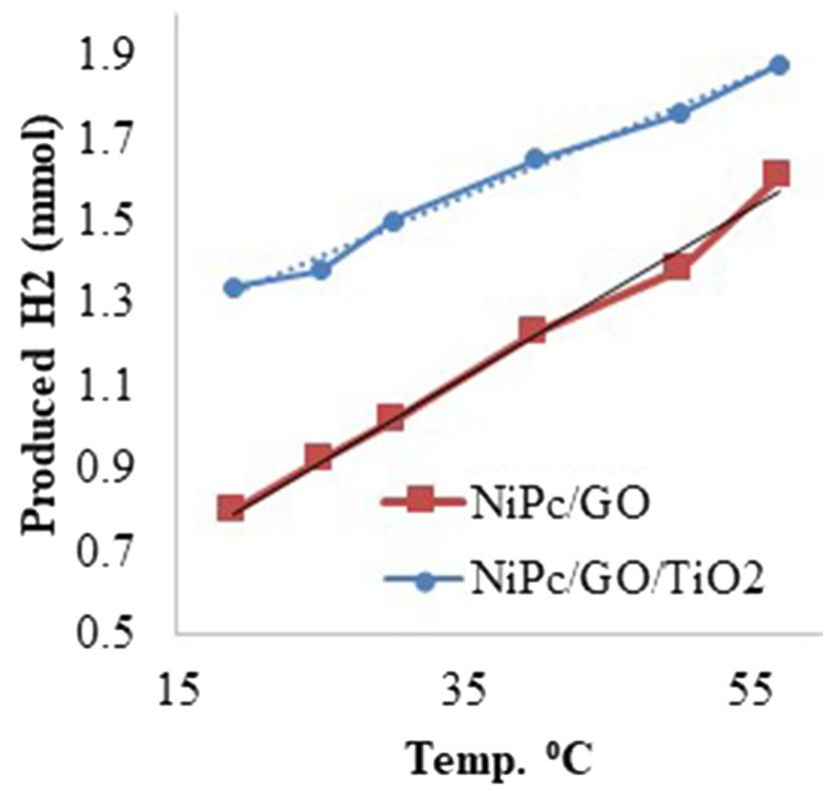
Temperature effect on NiPc@GO and NiPc@GO/TiO_2_.

The kinetic study was carried out on the reaction via investigation of temperature effect on the reaction assuming that the reaction is not diffusion-limited below 57 °C. Temperature had an important influence on the catalytic activity of NiPc@GO/TiO_2_ and NiPc@GO, where a high temperature was beneficial for the dehydrogenation reaction (Fig. 7). Moreover, the activation energy (E_a_) was calculated to be 9.1 and 19.0 kJ/mol for the reaction by NiPc@GO/TiO_2_ and NiPc@GO, respectively. These values are considerably lower than most previously reported E_a_ values^13^.

Potential NiPc leaching into the mixture of FA degradation reaction was also analyzed with FAAS analysis. For this purpose, filtrate of the heterogeneous degradation reaction of FA after 1 h was passed from a syringe filter and then dissolved in HNO_3_. The FAAS analysis of sample evinced that the Ni concentration in the reaction mixture was less than the detection limit. This result indicates that virtually no NiPc leaches from NiPc@GO into the mixture. A hot filtration test was also performed on NiPc@GO by removing the catalyst from the reaction mixture after 10 min and monitoring the reaction progress. Under this condition, gas releasing was completely quenched confirming heterogeneously done the reaction. Finally, the catalyst stability was examined in the reaction mixture. For that, the light source was removed after 1 h and irradiation was performed again after 24 h. This cycle was repeated for 5 cycles with 1.38 mmol h^−1^ H_2_ was produced in each of them, which shows as expected TiO_2_, NiPc, and GO are stable in FA.

## Conclusion

NiPc@GO was prepared through synthesis of NiPc in the presence of GO, which uniform distribution of NiPc on GO was obtained. Superior photocatalytic activity was observed for NiPc@GO in the degradation reaction of formic acid under visible light toward production of H_2_. In addition, TiO_2_ improved NiPc@GO catalytic activity significantly. Results showed that TiO_2_ as a semiconductor, and NiPc as a semiconductor as well as photosensitizer contribute in this reaction. Additionally, GO by improving electron injection efficiency and retarding the charge recombination in semiconductors accelerate the reaction.

## Data Availability

All data generated or analysed during this study are included in this published article.

## References

[1] Staffell I (2019). The role of hydrogen and fuel cells in the global energy system. Energy Environ. Sci..

[2] Dutta S (2014). A review on production, storage of hydrogen and its utilization as an energy resource. J. Ind. Eng. Chem..

[3] Fellay C, Yan N, Dyson PJ, Laurenczy G (2009). Selective formic acid decomposition for high-pressure hydrogen generation: A mechanistic study. Chem. A Eur. J..

[4] Li X, Ma X, Shi F, Deng Y (2010). Hydrogen generation from formic acid decomposition with a ruthenium catalyst promoted by functionalized ionic liquids. Chemsuschem.

[5] Czaun M (2011). Hydrogen generation from formic acid decomposition by ruthenium carbonyl complexes. Tetraruthenium dodecacarbonyl tetrahydride as an active intermediate. Chemsuschem.

[6] Zhang YJ, Zhang L (2009). Photocatalytic degradation of formic acid with simultaneous production of hydrogen over Pt and Ru-loaded CdS/Al-HMS photocatalysts. Desalination.

[7] Santos JL (2021). Functionalized biochars as supports for Pd/C catalysts for efficient hydrogen production from formic acid. Appl. Catal. B Environ..

[8] Jiang Y (2020). Facile synthesis of AuPd nanoparticles anchored on TiO_2_ nanosheets for efficient dehydrogenation of formic acid. Nanotechnology.

[9] Tamarany R (2021). Formic acid dehydrogenation over PdNi alloys supported on N-doped carbon: Synergistic effect of Pd–Ni alloying on hydrogen release. Phys. Chem. Chem. Phys..

[10] Seger B, Lu GQM, Wang L (2012). Electrical power and hydrogen production from a photo-fuel cell using formic acid and other single-carbon organics. J. Mater. Chem..

[11] Kiliç EÖ, Koparal AS, Öğütveren ÜB (2009). Hydrogen production by electrochemical decomposition of formic acid via solid polymer electrolyte. Fuel Process. Technol..

[12] Gazsi A, Schubert G, Pusztai P, Solymosi F (2013). Photocatalytic decomposition of formic acid and methyl formate on TiO_2_ doped with N and promoted with Au. Production of H_2_. Int. J. Hydrogen Energy.

[13] Du S, Zhang C, Jiang P, Leng Y (2019). Palladium nanoparticles immobilized on nitride carbon-coated mesoporous tungsten oxide for formic acid dehydrogenation. ACS Appl. Nano Mater..

[14] Gregory P (2012). Industrial applications of phthalocyanines. J. Porphyr. Phthalocyanines.

[15] Perez EF, Kubota LT, Tanaka AA, Neto GD (1998). Anodic oxidation of cysteine catalysed by nickel tetrasulphonated phthalocyanine immobilized on silica gel modified with titanium(IV) oxide. Electochim. Acta.

[16] Hassan SA, Zahran AA, Yehia FZ (2002). A supported nickel phthalocyanine complex as a selective catalyst for the production of styrene. Adsorp. Sci. Technol..

[17] Ma W, Wu J, Shen C, Tang H, Pan M (2008). Nickel phthalocyanine-tetrasulfonic acid as a promoter of methanol electro-oxidation on Pt/C catalyst. J. Appl. Electrochem..

[18] Zhang X (2020). Molecular engineering of dispersed nickel phthalocyanines on carbon nanotubes for selective CO_2_ reduction. Nat. Energy.

[19] Tiwari B, Noori MT, Ghangrekar M (2018). Enhancing performance of microbial fuel cell treating distillery wastewater using carbon supported Nickel-phthalocyanine/MnO_x_ as novel cathode catalyst. MRS Adv..

[20] Mousali E, Zanjanchi MA (2020). Loading of nickel phthalocyanine onto functionalized mesoporous KIT-6 solid support: an efficient visible photocatalyst for the degradation of 2,4-dichlorophenol. React. Kinet. Mech. Catal..

[21] Yuan Y-J (2016). Neutral nickel (II) phthalocyanine as a stable catalyst for visible-light-driven hydrogen evolution from water. Dalton Trans..

[22] Shaabani A, Keshipour S, Hamidzad M, Shaabani S (2014). Cobalt (II) supported on ethylenediamine-functionalized nanocellulose as an efficient catalyst for room temperature aerobic oxidation of alcohols. J. Mol. Catal. A Chem..

[23] Kaeffer N, Massin J, Lebrun C, Renault O, Chavarot-Kerlidou M, Artero V (2016). Covalent design for dye-sensitized H_2_-evolving photocathodes based on a cobalt diimine-dioxime catalyst. J. Am. Chem. Soc..

[24] Li F, Fan K, Xu B, Gabrielsson E, Daniel Q, Li L, Sun L (2015). An Organic dye-sensitized tandem photoelectrochemical cell for light driven water splitting. J. Am. Chem. Soc..

[25] Pati PB, Zhang L, Philippe B, Fernández-Terán R, Ahmadi S, Tian L, Rensmo H, Hammarström L, Tian H (2017). Insights into the mechanism of a covalently-linked organic dye-cobaloxime catalyst system for dye sensitized solar fuel devices. Chemsuschem.

[26] Qurashi A, Zhang Z, Asif M, Yamazaki T (2015). Template-less surfactant-free hydrothermal synthesis NiO nanoflowers and their photoelectrochemical hydrogen production. Int. J. Hydrogen Energy.

[27] Al-Azmi A, Keshipour S (2019). Cross-linked chitosan aerogel modified with Pd (II)/phthalocyanine: Synthesis, characterization, and catalytic application. Sci. Rep..

[28] Bahluli R, Keshipour S (2019). Microcrystalline cellulose modified with Fe (II)–and Ni (II)–phthalocyanines: Syntheses, characterizations, and catalytic applications. Polyhedron.

[29] Zhang X (2014). Highly asymmetric phthalocyanine as a sensitizer of graphitic carbon nitride for extremely efficient photocatalytic H_2_ production under near-infrared light. ACS Catal..

[30] Keshipour S, Al-Azmi A (2020). Synthesis and catalytic application of Pd/PdO/Fe_3_O_4_@polymer-like graphene quantum dots. Appl. Organomet. Chem..

[31] Dong Y (2012). Blue luminescent graphene quantum dots and graphene oxide prepared by tuning the carbonization degree of citric acid. Carbon.

[32] Keshipour S, Kulaei M, Ahour F (2019). Graphene Oxide Nano-Sheets-Supported Co(II)-d-Penicillamine as a Green and Highly Selective Catalyst for Epoxidation of Styrene. Iran. J. Sci. Technol. Trans. Sci..

[33] Manwar NR (2016). Ceria supported Pt/PtO-nanostructures: Efficient photocatalyst for sacrificial donor assisted hydrogen generation under visible-NIR light irradiation. ACS Sustain. Chem. Eng..

[34] Lever A (1981). Charge-transfer spectra of metallophthalocyanines: correlation with electrode potentials. J. Am. Chem. Soc..

[35] Cataldo F (1997). Synthesis and study of electronic spectra of planar polymeric phthalocyanines. Dyes Pigments.

[36] Martins PM (2018). TiO_2_/graphene and TiO_2_/graphene oxide nanocomposites for photocatalytic applications: A computer modeling and experimental study. Compos. B Eng..

[37] Eley DD (1948). Phthalocyanines as semiconductors. Nature.

